# The Excretion of Formaldehyde by the Kidneys of Patients Taking Urotropin

**Published:** 1913-03

**Authors:** 


THE EXCRETION OF FORMALDEHYDE BY THE KIDNEYS OF PATIENTS TAKING UROTROPIN.—L'Esperance (Boston *Med. and Surg. Jour.*, October 24, 1912), states that formaldehyde appears in the urine in only about 52 per cent. of the patients taking urotropin, regardless of the size of the dose administered. It is of great importance that the practitioner should know whether the urotropin is having the desired effect in typhoid, infections of the bladder, etc., and for this purpose Burnam's test seems reliable. The ordinary tests are too delicate.

To be of value, urotropin must be present in one part to 5000 or 6000. The duration of excretion is four to six hours. The best intervals for testing for its presence are, one-half, one hour or an hour and a half after administration. Burnam's test:

To 10 c. c. of urine in a test tube at body temperature is added:

1. Of solution phenylhydrazine HCl, 0.5% gtts. 3.
2. Solution sodium nitroprusside, 5% gtts. 3.
3. Of a saturated solution of sodium hydrate, a few drops are poured along the side of the test tube.

Positive.—A deep purplish-black color quickly changing to a dark green; gradually to a lighter shade and finally to a pale yellow.

Negative.—A reddish color, gradually changing to a light yellow.

The reaction of the urine is of no importance and the increase of dosage does not affect the excretion of urotropin in negative urines. Also, alkalies taken with or in combination with urotropin, have no effect on the excretion.

